# The Evolving Role of Regional Anaesthesia in Organ Transplantation: Raising the Bar in Perioperative Outcomes

**DOI:** 10.4274/TJAR.2026.262860

**Published:** 2026-08-28

**Authors:** Serkan Tulgar, Alper Kılıçaslan, Özlem Selvi Can, Zekeriyya Alanoğlu

**Affiliations:** 1 Bahçeşehir University Faculty of Medicine, Göztepe Medical Park Hospital, Department of Anaesthesiology and Reanimation, İstanbul, Türkiye; 2 Necmettin Erbakan University Faculty of Medicine, Department of Anaesthesiology and Reanimation, Konya, Türkiye; 3 Ankara University Faculty of Medicine, Department of Anaesthesiology and Reanimation, Ankara, Türkiye; 4 Washington University in St. Louis, Faculty of Medicine, Department of Anaesthesiology, St. Louis, United States

Only a few decades ago, liver and kidney transplantation were procedures performed in a limited number of highly specialized centers by exceptionally skilled surgeons and anaesthesiologists. At a time when case volumes were low and perioperative management remained highly demanding, the primary goal was straightforward: to ensure the survival of both the recipient and, in living donor transplantation, the donor, while minimizing major perioperative complications. As surgical techniques became more refined and transplant procedures gradually shifted toward less invasive approaches, the priorities of the perioperative team also began to change. Mortality and major morbidity were no longer the only endpoints. Attention gradually shifted to the quality of postoperative recovery. Effective analgesia, prevention of postoperative nausea and vomiting, and early mobilization became the main targets of perioperative care. Interestingly, all three were closely linked. Better pain control facilitated early mobilization, while reduced opioid consumption translated into less postoperative nausea and vomiting.

It was therefore natural for anaesthesiologists, as the physicians responsible for perioperative care, to seek opioid-sparing analgesic strategies. Interfascial plane blocks arrived at the right time. Although they may not provide the same density of blockade as neuraxial techniques, they may avoid some of the limitations and risks associated with neuraxial analgesia. More importantly, when incorporated into a multimodal analgesic regimen, they have the potential to provide meaningful clinical benefits while reducing reliance on opioids. Reflecting the growing interest in interfascial plane blocks, this issue features a letter to the editor by Özenet al., describing the combined use of ultrasound-guided rectointercostal and transversalis fascia plane blocks for postoperative analgesia in a pediatric renal transplant recipient, alongside a comprehensive narrative review by De Cassai et al., discussing regional anaesthesia techniques for both donors and recipients in liver and kidney transplantation and the role of interfascial plane blocks in postoperative pain management.

These articles also remind us that regional anaesthesia is far more than the technical execution of a block learned through a traditional apprenticeship model. Selecting the appropriate technique requires an understanding of the surgical procedure itself, the differences between donor and recipient operations, the tissues involved, and the mechanisms underlying postoperative pain. This knowledge must then be integrated with detailed anatomy and the pharmacology and pharmacokinetics of local anaesthetics and adjuvant drugs. It is this combination of surgical insight, anatomical knowledge, and sound clinical judgment that continues to advance the field of regional anaesthesia.

The value of this approach becomes even more evident in the most challenging clinical scenarios. In this issue, Akin et al. present a case report describing the combined use of M-TAPA and pecto-intercostal fascial block for analgesia management in the intensive care unit in a high-risk patient who underwent simultaneous liver transplantation and coronary artery bypass grafting surgery. Such cases illustrate that interfascial plane blocks are not limited to routine postoperative pain control; when applied with the same anatomical and clinical rigor, they can also serve as valuable tools in the analgesic management of complex, high-risk patients in the intensive care setting.

We believe that the future of regional anaesthesia lies not in asking whether a block has never been used for a particular operation or whether a previously unexplored fascial plane can be injected, but in selecting the right technique for the right patient, with the right indication, based on a sound understanding of surgical anatomy and pain mechanisms. We are proud to consider and publish manuscripts that contribute to this rational, patient-centered evolution of regional anaesthesia.
